# Flow-encoded raster line scanning (FERAL) of the peripheral arteries

**DOI:** 10.1186/1532-429X-13-S1-P383

**Published:** 2011-02-02

**Authors:** Robert R Edelman, Erik Offerman, Chris Glielmi, Eugene Dunkle, Ioannis Koktzoglou

**Affiliations:** 1 NorthShore University HealthSystem, Evanston, IL, USA; 2 Siemens Healthcare, Chicago, IL, USA

## Introduction

Peripheral arterial disease (PAD) is a debilitating disorder. Imaging evaluation is often performed with MRA or CTA, which show anatomic features of a stenosis rather than the hemodynamic abnormalities which produce the symptoms..

## Purpose

This study tested the feasibility of evaluating hemodynamic function over the entire peripheral arterial system using Flow-Encoded RAster Line scanning (FERAL).

## Methods

Imaging was performed on a 1.5T Siemens Avanto system. FERAL MRA was acquired as a series of real-time, time-resolved (20 ms/frame) transversal 3mm-thick, one-dimensional phase contrast (paired rephased/dephased) acquisitions, with each cine series spanning a single R-R interval. Overlapping line scan data are reprojected into the coronal plane for cine viewing and quantitative evaluation. Two data acquisition approaches were tested to minimize background phase shifts (which otherwise would cause inaccurate velocity measurements): (1) RACE-like technique with 90 degree flip angle; (2) a subtraction technique using the difference of unsaturated and flow-suppressed data and reduced flip angle of 25 degrees. Fifteen contiguous table positions were typically used to span the peripheral arteries from calf through supra-renal abdominal aorta.

## Results

**Figure 1:**
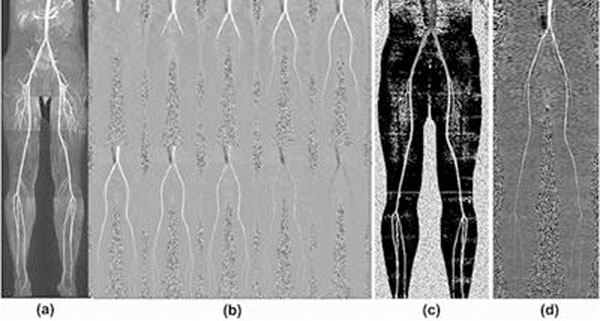
(a)MRA, b)FERAL, c)time-to-peak map, d)mean velocity map.

Testing in a constant flow phantom revealed excellent correlation (r2=0.999) between mean flow velocities as measured by FERAL and 2D phase contrast MRI Next, an IRB-approved study of FERAL MRA was performed in healthy subjects and ten patients with PAD. Strong agreement between FERAL and 2D PC was obtained for mean arterial velocity (intraclass correlation coefficient = .830; P < 0.01) and for the time of peak blood flow (intraclass correlation coefficient = .801; P < 0.01). The RACE approach showed better vessel conspicuity, but the subtraction technique with smaller flip angle better depicted triphasic flow. In healthy subjects, smooth, bilaterally symmetrical progression of flow was visualized from the supra-renal aorta to the ankles (Fig. 1). Abnormal flow patterns including delayed pulse wave and collateral flow were depicted. For instance, in a patient with severe PAD and resultant ulceration of the right foot, FERAL (but not MRA) demonstrated the hemodynamic consequences of a vessel occlusion, including unilaterally decreased mean flow velocity and slower pulse wave.

## Conclusions

FERAL MRA permits the evaluation of hemodynamic abnormalities throughout the peripheral arterial system in less than ten minutes. Moreover, the images are readily co-registered with standard MRA and, unlike DUS, the technique is largely operator-independent. The combination of luminographic (MRA)and hemodynamic (FERAL) information offers potential benefit for managing patients with PAD.

